# Effectiveness of Cognitive Behavioural Therapy for Insomnia (CBT‐I) in Individuals With Neurodevelopmental Conditions: A Systematic Review

**DOI:** 10.1111/jsr.70058

**Published:** 2025-04-03

**Authors:** Maja Cullen, Stephanie McCrory, Gemma Hooman, Megan Coyle, Leanne Fleming

**Affiliations:** ^1^ Department of Psychological Sciences and Health, Faculty of Humanities and Social Sciences University of Strathclyde Glasgow Scotland UK

**Keywords:** ADHD, ASD, CBT‐I, insomnia, neurodivergence, sleep

## Abstract

Cognitive Behavioural Therapy for insomnia (CBT‐I) is the recommended first‐line treatment for chronic insomnia disorder in diverse populations with co‐occurring conditions. However, individuals with neurodevelopmental conditions are frequently excluded from CBT‐I research, despite the high prevalence of sleep problems in this population. The present systematic review and narrative synthesis investigates the effectiveness of CBT‐I in individuals with neurodevelopmental conditions. A literature search was completed in February 2024 across five databases. Of 1988 unique entries, 8 studies from 5 countries met all inclusion criteria, amounting to a combined sample size of 598 participants (male = 75.92%, age range = 4–68). Five interventions were delivered to children and adolescents (*M* = 8.7 years ±1.46), whilst 3 were delivered to adults (*M* = 35.78 years ±5.71). All interventions included at least 2 CBT‐I components but were diverse in content and format. Two randomised controlled trials and six before‐after studies suggested significant short‐term effectiveness of CBT‐I in individuals with Autism Spectrum Disorder (ASD) and/or Attention‐Deficit/Hyperactivity Disorder (ADHD). Improvements in the severity of condition‐specific characteristics were observed in some studies. Still, findings were inconsistent and mostly not maintained at follow‐up. The quality of included articles was moderate due to small sample sizes, lack of blinding and deviations from the intended intervention. This variability may be explained by a lack of guidance on conducting CBT‐I in this population. Hence, there is a need for further rigorous research and updated reviews to inform the implementation of CBT‐I protocols for those with insomnia and neurodevelopmental conditions.

## 1 Introduction

Adequate, restorative sleep that is sufficient in quantity and quality is essential for physiological and psychological wellbeing (Itani et al. 2017; Scott et al. 2021; Wardle‐Pinkston et al. 2019). However, estimates suggest that up to a third of the adult population experience sleep problems (Chunnan et al. 2022; Kerkhof 2017; Ohayon 2011). Similar observations were made in children and adolescent samples (Barclay et al. 2015; Fricke‐Oerkermann et al. 2007). The most prevalent sleep disorder is insomnia disorder (ID), which affects approximately 10% of adults (Baglioni et al. 2020) and up to a fifth of typically developing children (Barclay et al. 2015; Falch‐Madsen et al. 2020), although estimates vary considerably depending on co‐occurring mental and physical health conditions (e.g., Chunnan et al. 2022; Smith et al. 2023). ID is a recognised mental health disorder, characterised by day‐ and night‐time symptoms that are present at least three nights per week, for a minimum duration of 3 months (ICSD‐3‐TR [American Academy of Sleep Medicine 2023]; DSM‐V [American Psychiatric Association 2013]; ICD‐11 [World Health Organization 2019]). Night‐time symptoms comprise difficulties initiating and/or maintaining sleep as well as early morning awakenings (EMA). Daytime symptoms include fatigue, mood disturbances (e.g., heightened irritability and aggression), attention and concentration deficits and impaired daily functioning.

The recommended first‐line treatment for ID in adults is Cognitive Behavioural Therapy for insomnia (CBT‐I) (Edinger et al. 2021; Riemann et al. 2023). CBT‐I is a multicomponent approach that targets maladaptive cognitions (e.g., counterproductive thought patterns, nighttime rumination, racing thoughts) and behaviours (e.g., daytime napping, staying in bed when awake, extending time in bed). Multi‐component CBT‐I includes Cognitive Therapy, Stimulus Control Therapy (SCT), Sleep Restriction Therapy (SRT), relaxation techniques and sleep hygiene. However, recent developments in ID research have demonstrated the effectiveness of single‐component treatment approaches, indicating that SCT, SRT and relaxation are the most effective (Edinger et al. 2021). Cognitive Therapy and sleep hygiene are not recommended as single‐component treatments due to insufficient evidence of their effectiveness (Edinger et al. 2021; Irish et al. 2015). Similarly, there is insufficient evidence of the effectiveness of other sleep interventions, such as intensive sleep retraining or mindfulness therapies, as treatments for ID.

CBT‐I can be delivered in various formats, such as individual (one‐to‐one) sessions, group sessions, face‐to‐face, or digitally (dCBT‐I) using websites and smartphone apps (Riemann et al. 2023). However, a recent meta‐analysis found that synchronous CBT‐I interventions yield stronger effects than asynchronous digital ones and should be prioritised whenever possible (Simon et al. 2023). CBT‐I is typically delivered over four to eight sessions, poses few side effects compared to pharmacological insomnia treatments and provides long‐term improvements to sleep (Jernelöv et al. 2022; Riemann et al. 2023).

The usefulness of CBT‐I has been assessed favourably across a variety of populations, including those with psychiatric and/or medical co‐morbidities (Hertenstein et al. 2022; Scott et al. 2021; Wu et al. 2015; Zhou et al. 2020). Whilst European Sleep Research Society guidelines (Riemann et al. 2023) only apply to adults with ID, and no similar guidance exists for paediatric populations, recent studies showcase successful implementation of CBT‐I protocols for children and adolescents (Dewald‐Kaufmann et al. 2019; Schlarb et al. 2016). However, little research attention has been paid to people of any age with neurodevelopmental conditions (NDC), such as Autism Spectrum Disorder (ASD) or Attention‐Deficit/Hyperactivity Disorder (ADHD), despite the fact that sleep problems are more common in people with NDC than in the neurotypical population (Angriman et al. 2015; Didden and Sigafoos 2001; Lugo et al. 2020; Robinson‐Shelton and Malow 2016). Prevalence estimates of troubled sleep within the NDC population differ significantly due to the variety of unique NDC, a lack of unitary definitions and poor assessment of sleep problems in this population (Owens 2007; Richdale and Baker 2014). However, sleep problem rates as high as 78% and 86% have been reported in adult and children samples, respectively (Van Veen et al. 2010; Liu et al. 2006). One study of children with ASD reported that 86% experience sleep disturbance and 56% meet criteria for ID (Liu et al. 2006). In a recent study of adults with ADHD, 63.9% of participants presented with at least one symptom of insomnia and over 44% met full diagnostic criteria for ID (Fadeuilhe et al. 2021). These data are concerning as insufficient, poor‐quality sleep in people with NDC is associated with poorer quality of life and intensified condition specific characteristics, such as larger communication deficits (Schreck et al. 2004; Tudor et al. 2012).

Despite these prevalence estimates, a limited number of studies have explored the effective treatment of sleep problems in NDC populations (e.g., Corkum et al. 2016; Hiscock et al. 2015; Yuge et al. 2020). Most research focusses on child and adolescent samples and utilises sleep hygiene and pharmacological treatments to improve sleep (e.g., Adkins et al. 2012; Blackmer and Feinstein 2016; Owens et al. 2010; Yuge et al. 2020). This is problematic as the safety of long‐term pharmacological treatment is unclear and there is an absence of guidelines on dosing regimens (Bruni et al. 2015; Givler et al. 2023). Moreover, the overwhelmingly positive impact of CBT‐I treatments justifies increased research attention for NDC populations of all ages. This systematic review addresses this evidence gap by examining the effectiveness of CBT‐I in individuals of all ages with NDC.

## 2 Methods

### 2.1 Reporting and Registration

The review is reported in concordance with the Preferred Reporting Items for Systematic Reviews and Meta‐Analyses (PRISMA) statement (Liberati et al. 2009). The full protocol for this systematic review was registered on PROSPERO (registration number CRD42024509225) and is publicly available (https://www.crd.york.ac.uk/prospero/display_record.php?ID=CRD42024509225).

### 2.2 Search Strategy

A search of the literature was completed in February 2024 using PsycINFO, Scopus, Medline and Cochrane Library databases. Additionally, the search engine Google Scholar was used to enable access to a broad variety of sources. Search terms included variations and synonyms of the sleep disorder under investigation, the target population and the relevant intervention (see Table 1). Boolean operators and truncation were used to minimise the likelihood of missing relevant research. Searches on Cochrane Library slightly deviated from the examples below due to the additional usage of parentheses and the proximity operator ‘NEXT’ in lieu of quotation marks for search terms involving more than one‐word phrases (see Appendix A for full search strategies). Study references were restricted to English publications on human samples of all ages. No publication date limitations were applied as this is the first review of its kind and every effort was made to be as comprehensive as possible. The search was aided by an experienced librarian with subject specialisms in social sciences and neurodiversity.

**TABLE 1:** Search terms used for literature search on PsycINFO, Scopus, Medline and Google Scholar.

Database used	Search terms used
PsyInfo, Scopus, Medline and Google Scholar	1. insomnia* OR ‘sleep disturbance*’ OR ‘sleep disorder*’ OR sleeplessness OR ‘sleep problem*’ OR ‘behavio* sleep disorder*’ OR ‘DIMS, Disorders of Initiating and Maintaining Sleep’ OR ‘Disorders of Initiating and Maintaining Sleep’ OR ‘Early Awakening’ OR ‘Insomnia Disorder*’, OR ‘sleep fragmentation’ OR ‘frequent awakening*’ OR ‘sleep onset’ OR ‘late insomnia’ OR ‘sleep maintenance’ OR ‘primary insomnia’
	2. neurodiver* OR ‘neurodevelopmental disorder*’ OR ‘neuropsychological disorder*’ OR ‘neuropsychology disabilit*’ OR ‘neurodisability’ OR ‘neurdevelopmental condition*’ OR ‘ASD’ OR Autis* OR ‘autism spectrum disorder*’ OR ASC OR ‘autism spectrum condition*’ OR ‘attention deficit hyperactivity disorder*’ OR ADHD OR ‘attention deficit disorder*’ OR ‘attention deficit*’ OR asperger* OR ‘intellectual disability*’ OR ‘learning difficult*’
	3. Intervention* OR CBT OR ‘cognitive behavio* therap*’ OR ‘cognitve therap*’ OR ‘behavio* intervention*’ OR ‘sleep restriction’ OR ‘bed restriction’ or ‘sleep hygiene’ OR ‘relaxation’ OR ‘sleep education’ OR ‘stimulus control’ OR ‘cognitive intervention*’ OR ‘psychoeducation’

### 2.3 Eligibility Criteria

To define the inclusion and exclusion criteria for this review, a PICO (Population, Intervention, Comparator and Outcome) model was used and included (i) people diagnosed with NDC and ID as the population of interest, (ii) multicomponent CBT‐I or a single evidence‐based component of CBT‐I as the intervention of interest, (iii) treatment as usual as the comparator and (iv) changes in insomnia severity (e.g., sleep onset latency [SOL], wakefulness after sleep onset [WASO], sleep efficiency [SE], total sleep time [TST], total wake time [TWT], EMA and satisfaction with sleep) as the primary outcome under investigation. Additionally, secondary outcomes were collected whenever possible and referred to mental wellbeing, severity of NDC characteristics and sleep habits. Accordingly, publications were eligible for inclusion if they: (i) included a sample of individuals diagnosed with ID and NDC. For ID, diagnosis was based on the use of a recognised diagnostic classification manual (e.g., DSM‐III, DSM‐IV or DSM‐V [American Psychiatric Association 1980, 1994, 2013]; ICSD‐3, ICSD‐3‐TR [American Academy of Sleep Medicine 2014, 2023]) or a clinical interview and/or validated sleep questionnaires (e.g., SLEEP‐50 [Spoormaker et al. 2005]) and sleep diaries. For NDC (e.g., ASD, ADHD, Cerebral Palsy, Foetal Alcohol Spectrum Disorders, Fragile X Syndrome or Down's Syndrome), diagnosis was based on the use of a recognised diagnostic classification manual (e.g., DMS‐III, DSM‐IV or DSM‐V [American Psychiatric Association 1980, 1994, 2013]; ICD‐9, ICD‐10 or ICD‐11 [World Health Organization; 1975, 2016, 2019]) and/or a standardised diagnostic tool (e.g., Autism Diagnostic Observation Schedule (ADOS) [Lord et al. 2000]) and (ii) the applied intervention included at least one single evidence‐based component of CBT‐I (e.g., SCT, SRT, relaxation) based on recommendations in Edinger et al. (2021) and Riemann et al. (2023). Eligible study designs included randomised controlled trials (RCTs), non‐randomised studies, cohort studies, pilot studies, feasibility studies and pre‐post studies. Case studies were excluded due to their low informative value. Qualitative, quantitative and mixed methods research was considered at the screening stage.

Studies were excluded if participants presented with a sleep disorder other than ID or if the effects of the intervention on ID were not discernible from the effects of the intervention on a co‐presenting sleep disorder. Additionally, interventions that only used Cognitive Therapy, sleep hygiene or sleep education as the sole CBT‐I component were excluded due to recommendations against their usefulness as single‐component therapies (Edinger et al. 2021).

### 2.4 Study Selection

Results of the literature search were uploaded to the screening platform Rayyan (https://www.rayyan.ai/). Eligible studies were initially screened based on abstracts, followed by a full‐text review. The same inclusion and exclusion criteria were used at both stages of screening. When abstracts did not directly oppose the applied PICO framework, the entire article was reviewed to establish fit. Each study was independently assessed for eligibility by three researchers (MC, GH and MC). 92% agreement was achieved between researchers at the abstract screening stage and 90% agreement during full‐text screening. In case of discrepancies, consensus was reached by majority opinion. When information was unclear or inaccessible, authors were contacted.

### 2.5 Data Extraction

Data of included studies were extracted independently by three researchers (MC, GH and MC) and then reviewed by other researchers (SM and LF). Data extracted included study characteristics (i.e., authors, year of publication, country of publication, study aim, study design, recruitment and sampling strategy, inclusion and exclusion criteria and participant screening procedures), population characteristics (i.e., sample size, drop‐outs, age, sex, ethnicity, socioeconomic status, psychiatric co‐morbidities, insomnia diagnosis, NDC diagnosis and parental support with intervention delivery) and intervention characteristics (i.e., setting, mode of delivery, format, facilitator, length of sessions, number of sessions, intensity of the intervention, CBT‐I components used, additional components used and adaptations for NDC).

### 2.6 Data Analysis

Our initial intention was to conduct a meta‐analysis. However, data extraction revealed that most studies were small‐scale pilots of moderate quality with diverse intervention protocols and outcomes, resulting in a high degree of heterogeneity. Therefore, a narrative synthesis was selected as a more appropriate method to critically examine findings and risk of bias across studies. Based on guidance from the Economic and Social Research Council (Popay et al. 2006), the present narrative synthesis comprised the development of a preliminary synthesis, an exploration of the commonalities and differences across and within the included data sets and an evaluation of the synthesis' robustness through a continuous exchange within the research team. Specific tools that were implemented in this process were textual descriptions of the individual studies, tabulation of study characteristics and results and a critical reflection on the synthesis process.

### 2.7 Quality Assessment of Individual Studies

Quality assessments for RCTs were undertaken using the Risk of Bias 2 tool (RoB 2 [Sterne et al. 2019]), a comprehensive instrument to establish the risk of bias in RCTs. Specifically, it assesses five domains of bias: bias arising from the randomisation process, bias due to deviations from the intended intervention, bias due to missing outcome data, bias in measurement of the outcome and bias in selection of the reported result. RCTs may receive a rating of low risk of bias, some concerns, or high risk of bias grounded in responses to hierarchically organised signalling questions.

The quality assessment for all remaining studies was undertaken using the National Heart, Lung and Blood Institute (NHLBI 2021) tool for pre‐post studies without control groups, which ranks the quality of before‐after studies from poor to fair to good. The tool is concise in its nature and comprises 12 individual questions that may be answered with ‘yes’, ‘no’ or ‘other’.

Neither risk of bias tools provides numerical outcomes, and both are explicitly dependent on reviewers' judgement. Quality assessment of all studies was performed separately by three researchers (MC, GH and MC) with the majority opinion deciding the final verdict.

## 3 Results

### 3.1 Search Results

**FIGURE 1:**
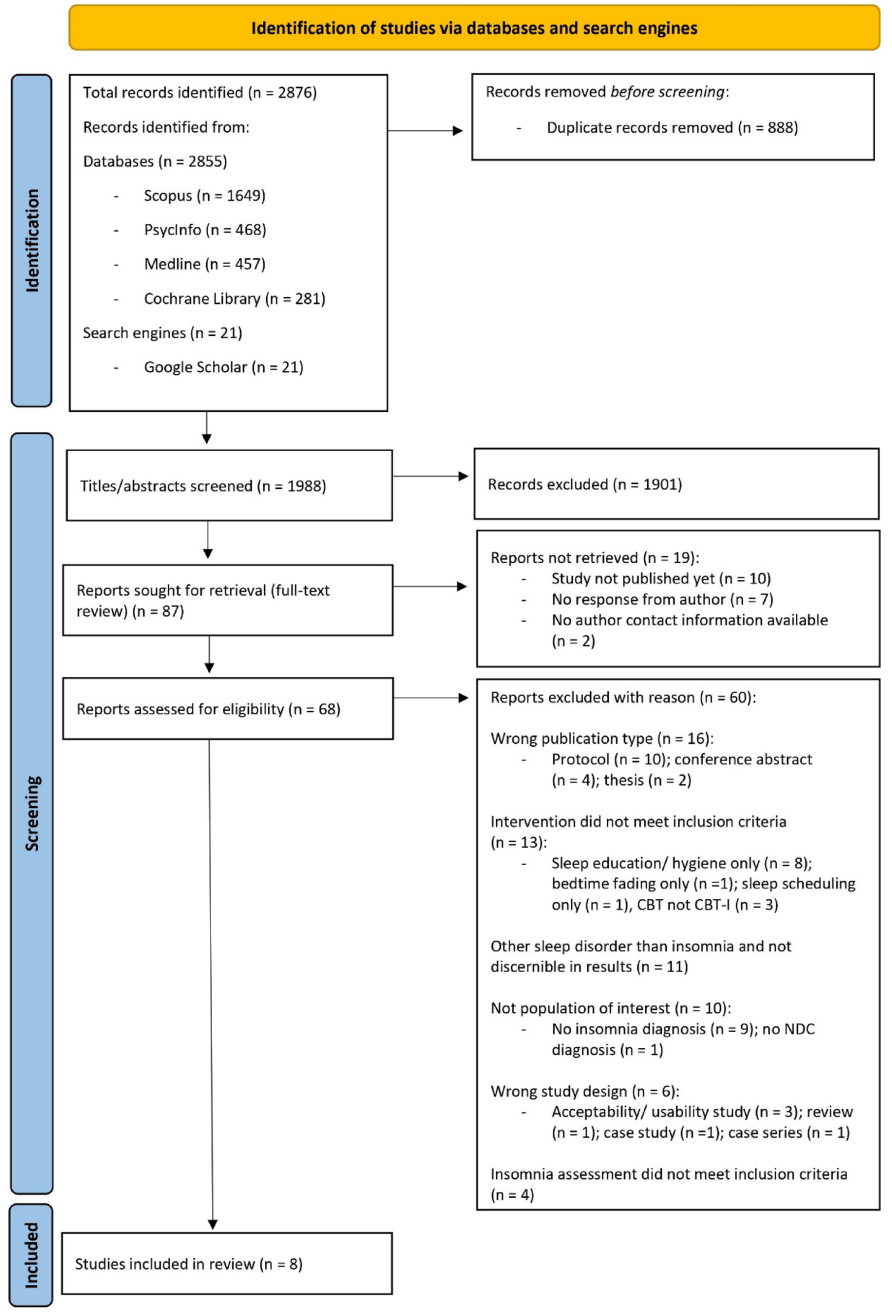
PRISMA flow diagram of study selection.

The literature search yielded 2876 articles. 888 of those were duplicates and subsequently removed. Accordingly, 1988 studies were included in the title/abstract screening. At this stage, most papers (*n* = 1901) were excluded due to encompassing populations, interventions or outcomes that did not align with the PICO framework for this review. Eighty‐seven papers were eligible for full‐text screening and subsequently sought for retrieval. For articles that could not be retrieved and/or if study details were unclear, authors were approached via email. Ten of the 87 entries were not published at the time of writing (November 2024). A further nine studies could not be accessed due to no reply from authors (who were contacted multiple times over a 9‐month period) or because no author contact information was available. Accordingly, 68 articles were included for full‐text screening, and 8 papers met all inclusion criteria. The main reasons for studies being excluded were (1) wrong publication type (*n* = 16), (2) interventions not meeting inclusion criteria (*n* = 13), (3) including participants with sleep disorders other than ID that could not be separated in results (*n* = 11), (4) interventions consisting of samples with no ID or NDC (*n* = 10), (5) inappropriate study designs for the purposes of this review (*n* = 6) and (6) ID assessments not aligning with the review's inclusion criteria (*n* = 4). Details for exclusion of all studies are provided in Figure 1.

### 3.2 Study Characteristics

The eight included studies spanned five countries, namely, the USA (*n* = 2), Sweden (*n* = 2), Australia (*n* = 2), Japan (*n* = 1) and Italy (*n* = 1). Study designs were predominantly pre‐post pilot studies (*n* = 6). Only one study was a RCT (Cortesi et al. 2012) and one study adopted a cluster‐RCT design (Hiscock et al. 2019). The oldest included study was published in 2012 (Cortesi et al. 2012) and the most recent study was published in 2023 (Lawson et al. 2023).

### 3.3 Sample Characteristics

The combined sample size of all studies was 598 (6–361 participants). Three hundred of the 598 participants were allocated to a CBT‐I sleep intervention, whilst the remaining 298 were assigned to control groups across the two randomised trials (Cortesi et al. 2012; Hiscock et al. 2019). Five studies consisted of children and adolescent samples (weighted mean age = 8.7 years ±1.46, *n* = 561, range = 4–17) (Cortesi et al. 2012; Georén et al. 2022; Hiscock et al. 2019; McCrae et al. 2020, 2021). The remaining three studies included adult‐only samples (*n* = 37, weighted mean age = 35.78 years ±5.71, range = 19–68) (Ishii et al. 2021; Jernelöv et al. 2019; Lawson et al. 2023). 75.92% of all participants identified as males (*n* = 454).

All samples consisted exclusively of participants with a diagnosed NDC. Five studies specifically focussed on people with ASD (Cortesi et al. 2012; Georén et al. 2022; Lawson et al. 2023; McCrae et al. 2020, 2021), two studies focussed on people with ADHD (Hiscock et al. 2019; Jernelöv et al. 2019) and one study designed a transdiagnostic intervention to treat ID in people with ASD and/or ADHD (Ishii et al. 2021). However, co‐occurrences of ASD and ADHD were commonly reported across all studies. Hence, of all participants, 166 were diagnosed with both ASD and ADHD, 190 with ASD only and 242 with ADHD only. No other NDC were disclosed. Notably, the three studies with adult participants did not report on possible co‐occurrences of intellectual disability within their samples (Ishii et al. 2021; Jernelöv et al. 2019; Lawson et al. 2023), but all five studies with children and adolescents had intellectual disability as an exclusion criterion (Cortesi et al. 2012; Georén et al. 2022; Hiscock et al. 2019; McCrae et al. 2020, 2021).

The most common psychiatric co‐morbidities reported were anxiety and depression. Across all 598 participants, 30 individuals reported experiencing anxiety and 19 reported experiencing depression. However, reporting of mental health problems was inconsistent across studies and entirely absent in three of them (Cortesi et al. 2012; Ishii et al. 2021; Lawson et al. 2023). Of the three studies that reported on ethnicity (Cortesi et al. 2012; McCrae et al. 2020, 2021), approximately 95% of participants were White or Caucasian.

### 3.4 Assessment of Insomnia and Neurodevelopmental Disorders

Insomnia was assessed in a variety of ways using self‐reports, validated questionnaires, diagnostic manuals, actigraphy data and various combinations of these. Notably, only four studies specifically utilised criteria outlined in diagnostic manuals (i.e., ICSD‐3 [American Academy of Sleep Medicine 2014]; DSM‐V [American Psychiatric Association, 2014]; ICD‐10 [World Health Organization 2016]) to assess sleep disorder status (Georén et al. 2022; Hiscock et al. 2019; McCrae et al. 2020, 2021). Three other studies (Ishii et al. 2021; Jernelöv et al. 2019; Lawson et al. 2023) relied on cut‐off scores on validated sleep questionnaires including the Insomnia Severity Index (ISI) (Morin 1993) and the SLEEP‐50 (Spoormaker et al. 2005).

Assessment of NDC was less variable and most studies requested a diagnosis established by diagnostic manuals (e.g., DSM‐IV, DSM‐5 [American Psychiatric Association 1994, 2013]) or diagnostic interviews/observations. To meet ASD inclusion criteria, studies largely relied on diagnoses based on different versions of the ADOS (Lord et al. 2000) and/or the Autism Diagnostic Interview—Revised (ADI‐R) (Rutter et al. 2003). To meet ADHD inclusion criteria, most studies relied on diagnoses based on the DSM‐V (American Psychiatric Association 2013).

### 3.5 Intervention Characteristics

Interventions varied in their implementation of CBT‐I components, format, mode of delivery, setting and duration. Three studies incorporated all five core CBT‐I components, namely, SRT, SCT, sleep hygiene, relaxation and Cognitive Therapy (Georén et al. 2022; McCrae et al. 2020, 2021). Two studies implemented four CBT‐I components (Jernelöv et al. 2019; Lawson et al. 2023), two studies included three CBT‐I components (Cortesi et al. 2012; Ishii et al. 2021) and one study included only two CBT‐I components (Hiscock et al. 2019). Other than Cortesi et al. (2012), all studies featured additional therapeutic components such as sleep compression (Jernelöv et al. 2019) or mindfulness (Jernelöv et al. 2019; Lawson et al. 2023). Homework assignments and workbooks were commonly integrated into treatment protocols (Georén et al. 2022; Jernelöv et al. 2019; McCrae et al. 2020, 2021).

The three adult interventions were all delivered in a group format, consisting of 2 to 10 participants (Ishii et al. 2021; Jernelöv et al. 2019; Lawson et al. 2023). The other five interventions were delivered individually to participants and their guardians. Most studies were at least partially conducted in person, with only Georén et al. (2022) providing asynchronous dCBT‐I using a website, and McCrae et al. (2021) delivering synchronous treatment sessions using a video platform. The reporting of intervention settings was often unclear (Hiscock et al. 2019; Ishii et al. 2021; Lawson et al. 2023; McCrae et al. 2020) but ranged from entirely at‐home (Georén et al. 2022; McCrae et al. 2021) to hospital outpatient clinics (Cortesi et al. 2012; Jernelöv et al. 2019). The number of sessions varied from 2 to 10 (*M* = 6.88 ± 2.71), and sessions lasted between 50 to 90 min (*M* = 68.57 ± 18.84). Intervention durations were estimated to fluctuate from a minimum of 2 to a maximum of 12 weeks (*M* = 7.25 ± 2.95), and interventions were predominantly delivered by experienced psychologists or therapists (Cortesi et al. 2012; Georén et al. 2022; Ishii et al. 2021; Jernelöv et al. 2019).

### 3.6 Intervention Adaptations

As there are currently no standardised protocols for the implementation of CBT‐I in populations with NDC, adaptations to the interventions were diverse and reliant on the researchers' expertise. Whilst some studies declared that their treatment was individually tailored in consideration of the participants' developmental characteristics, comprehensive information on these modifications was not specified (Cortesi et al. 2012; Ishii et al. 2021; Hiscock et al. 2019). Other studies provided more detail, stating that interventions were adapted by simplifying vocabulary (Georén et al. 2022), shortening sessions (Jernelöv et al. 2019), increasing repetition of materials and incorporating special interests into the treatment (McCrae et al. 2020, 2021). Lawson et al. (2023) explicitly consulted three adults with ASD in the design of their intervention to ensure that its content and language were appropriate for the target population (see Table 2 for full details).

**TABLE 2:** Study characteristics.

Author (year), country	Study design	Sample recruited (analysed)	Insomnia inclusion criteria	NDC inclusion criteria	Co‐morbidities	Intervention format	CBT‐I components	Additional components	Adaptations for NDC
Cortesi et al. (2012), Italy	RCT: CBT only, melatonin only, CBT and melatonin, placebo	*n* (total) = 160 (134) *n* (intervention group; IG) = 40 (33) Mean age [IG] (years) = 7.10 (SD = 0.70) Sex male [IG] (%): 83 Ethnicity [IG] (%): 100 White Caucasian	Mixed sleep onset and maintenance insomnia, defined as a sleep onset latency (SOL) and wake after sleep onset (WASO) > 30 min that occurred on ≥ 3 nights a week	Diagnosis of ASD from the DSM‐IV‐TR, confirmed by Autistic Diagnostic Interview‐ revised (ADI‐R) and Autism Diagnostic Observation Schedule‐generic (ADOS‐G)	N/R	One‐to‐one in person, 4 × 50 min CBT sessions + twice monthly individually tailored CBT sessions at an outpatient university clinic	Cognitive therapy Sleep hygiene Stimulus control	N/A	Individually tailored sessions, however, no details reported on specific adaptations
Georén et al. (2022), Sweden	Pre‐post within‐group pilot design	*n* = N/R (6) Mean age (years) = 15.50 (SD = 1.64) Sex male (%): 66.66 Ethnicity (%): N/R	Insomnia according to the ICD‐10 confirmed by an interview based on DSM‐V	Community based clinical consensus diagnosis of ASD using the ADOS 1st or 2nd edition	ADHD (*n* = 2), generalised anxiety disorder (*n* = 1), obsessive compulsive disorder (*n* = 2)	Self‐directed online intervention using a website at home; eight modules (approx. each 60 min to complete)	Cognitive therapy Sleep hygiene Stimulus control Sleep restriction Relaxation	Home assignments, support phone calls	Adaptations to vocabulary of modules' content to suit target population's needs
Hiscock et al. (2019), Australia	Cluster‐RCT: Behavioural inter‐vention, TAU	*n* (total) = 361 *n* (3 months) = 271 *n* (6 months) = 275 Mean age [IG] (years) = 9.60 (SD = 1.70) Sex male (%): 74.90 Ethnicity (%): N/R	Met the ICSD‐3 criteria for chronic insomnia	DSM‐V diagnostic criteria for ADHD based on ADHD rating scale IV/presence of characteristics for at least 6 months with cross‐sectional impairment	Internalising co‐morbidities (*n* = 116), externalising co‐morbidities (*n* = 83), ASD (*n* = 74)	Two one‐to‐one in‐person sessions + follow‐up support phone calls delivered by paediatricians or psychologists; location N/R	Sleep hygiene Relaxation	Limit setting (ignoring child protests and rewarding adherence to bedtime routine), information sheet for parents	Individual sleep plans; no further details reported
Ishii et al. (2021), Japan	Pre‐post within‐group pilot design	*n* (total) = 12 (10) Mean age (years) = 27.40 (SD = 5.60) Sex male (%): 100 Ethnicity (%): N/R	A score of ≥ 8 on the ISI + self‐reported nocturnal sleep problems and excessive daytime sleepiness	Diagnosis of suspected ADHD and/or ASD based on DSM‐V criteria	N/R	5 × 90‐min in‐person group sessions with two to five participants per group led by therapists/clinical psychologists; location N/R	Sleep hygiene Stimulus control Relaxation	Homework activities, limiting bright light exposure, sleep scheduling, regularity of sleep duration	Intervention designed in consideration of developmental characteristics of target population, but no details specified
Jernelöv et al. (2019), Sweden	Pre‐post within‐group pilot design	*n* = 19 (19) Mean age (years) = 37.0 (SD = 13.70) Sex male (%): 32 Ethnicity (%): N/R	SLEEP‐50 used to screen for sleep disorders	ADHD diagnosis confirmed by enrolment in specialist ADHD clinic and screening questionnaire	Depression (*n* = 15), anxiety (*n* = 13), panic attacks (*n* = 8), excessive worries (*n* = 8), post‐traumatic stress (*n* = 4), specific phobia (*n* = 4), intrusive thoughts (*n* = 4), psychosis (*n* = 1), manic episodes (*n* = 1), alcohol and/or substance abuse/addiction (*n* = 1), other (*n* = 3)	10 × 90‐min in‐person group sessions with 6 to 10 participants per group at a specialist outpatient ADHD clinic	Cognitive therapy Sleep hygiene Stimulus control Relaxation	Acceptance techniques, light scheduling, mindfulness, optional sleep compression	Adaptations made to suit needs of people with ADHD, including shortening sessions, offering scheduled telephone calls and extending treatment duration
Lawson et al. (2023), Australia	Concurrent group multiple baseline design; pre‐post pilot study	*n* = 12 (8) Mean age (years) = 43.38 (SD = 16.09) Sex male (%): 75 Ethnicity (%): N/R	A score of ≥ 8.00 on the ISI	Clinical diagnosis of ASD	N/R	4 × 90‐min in‐person group sessions with three to four participants per group led by a facilitator (N/R who facilitator is)	Cognitive therapy Sleep hygiene Stimulus control Sleep restriction	Acceptance for arousal reduction and emotional regulation, education about the effects of control and suppression on arousal rebound effects with thoughts, images and emotions, mindfulness, non‐judgmental awareness towards inner experiences and worry reduction techniques	Treatment designed under guidance of three adults with ASD to ensure suitability for target population; further adopted components from McCrae et al. (2020, 2021), but no details specified
McCrae et al. (2020), USA	Pre‐post within‐group pilot design	*n* = 17 (17) Mean age (years) = 8.76 (SD = 1.99) Sex male (%): 71 Ethnicity (%): 12 Hispanic 88 non‐Hispanic	Diagnosis of insomnia according to DSM‐V criteria	Diagnosed with ASD using ADI‐R obtained through clinical records at the Thompson Center ASD database	ADHD (*n* = 11), anxiety (*n* = 7), Asperger's syndrome (*n* = 2), developmental delays (*n* = 3), other (*n* = 2)	8 × 50‐min in‐person sessions led by doctoral students in counselling psychological or clinical psychology post‐doctoral fellow; location N/R	Cognitive therapy Sleep hygiene Stimulus control Sleep restriction Relaxation	Bedtime routine and parent management, co‐sleeping/parent fading out of the child's room, introducing child playtime circadian education, workbooks	Increased visual support and verbal explanations, higher rates of repetition and practice, consideration of sensory sensitivities, incorporation of unique special interests, adapted relaxation techniques, adapted cognitive strategies (e.g., cartoons to illustrate key concepts, concrete language), increased usage of behavioural strategies to address challenging behaviours
McCrae et al. (2021), USA	Pre‐post within‐group pilot design	*n* = 17 (17) Mean age (years) = 8.53 (SD = 1.70) Sex male (%): 82 Ethnicity (%): 100 non‐Hispanic	Diagnosis of insomnia according to DSM‐V criteria	Met DSM‐IV or DSM‐V criteria for ASD by using the ADOS and/or ADI‐R	ADHD (*n* = 14), anxiety (*n* = 9), Asperger's syndrome (*n* = 3), depression (*n* = 4), developmental delays (*n* = 5)	8 × 50‐min online at‐home sessions using videoconference platform Zoom; led by doctoral students in counselling psychology and school psychology	Cognitive therapy Sleep hygiene Stimulus control Sleep restriction Relaxation	Bedtime routine and parent management, co‐sleeping/parent fading out of child's room, circadian education, workbooks	Same as McCrae et al. (2020)

*Note: n* = number of participants.

Abbreviations: N/A, not applicable; N/R, not reported; TAU, treatment as usual.

### 3.7 Primary Outcomes

The primary outcomes of interest were changes in insomnia severity measured by various sleep parameters such as TST, SE, EMA, SOL, WASO and TWT. Outcomes were assessed objectively using actigraphy data and subjectively using sleep diaries and validated sleep questionnaires including the ISI (Morin 1993) or the Athens Insomnia Scale (AIS) (Soldatos et al. 2000). Generally, variables of interest were assessed at baseline, post‐treatment and follow‐up points ranging from 1 to 6 months. Cortesi et al. (2012) was the only study that did not administer a follow‐up assessment.

Overall, interventions appeared to offer short‐term effectiveness in reducing insomnia severity in children and adults with ASD and/or ADHD. These favourable changes in sleep quality and quantity were generally large when comparing pre‐intervention to post‐intervention data and applied to objective actigraphy results and subjective sleep diaries and questionnaires (Cortesi et al. 2012; Georén et al. 2022; Hiscock et al. 2019; Ishii et al. 2021; Jernelöv et al. 2019; McCrae et al. 2020, 2021). However, differences in objective and subjective perceptions of sleep were present when both were assessed. For example, Lawson et al. (2023) reported a significant reduction in ISI scores post‐intervention (*r* = −0.632, *p* = 0.011), but found no statistically significant changes in actigraphy outcomes. Similarly, participants in McCrae et al. (2020, 2021) self‐reported large pre‐ to post improvements in all sleep parameters, but these were not observed when exploring actigraphy data (e.g., SOL: −33 vs. −17 min, TWT: −46 vs. −32 min, TST: +52 vs. +34 min, SE: +8% vs. +5% [McCrae et al. 2021]).

Additionally, improvements in insomnia severity were largely not maintained at follow‐up (Hiscock et al. 2019; Ishii et al. 2021; Jernelöv et al. 2019; Lawson et al. 2023; McCrae et al. 2020, 2021). Hiscock et al. (2019) reported that their CBT‐I intervention was more effective in reducing sleep problems than treatment as usual. However, Cortesi et al. (2012) found that the greatest improvement in insomnia severity was observed in patients who received a combination of melatonin and CBT‐I. The melatonin only group was the next most improved, whilst the group receiving CBT‐I only was more successful in reducing insomnia severity than the placebo group who had little/no improvements (see Table 3 for more details).

**TABLE 3:** Key primary outcomes.

Author (year)	Primary outcome measures	Method of assessment	Assessment points	Main outcomes	Effect sizes
Cortesi et al. (2012)	SE, SOL, TST and WASO	Actigraphy and sleep diaries	Baseline, 12 weeks	Largest improvements found for CBT + melatonin group, followed by melatonin only group, followed by CBT only group, little or no improvement for placebo group: TST: COMB 22% improvement, CBT 9.31%, melatonin 17.31%, placebo 0.07% SOL: COMB 60.75%, CBT 22.54%, melatonin 44.33%, placebo 0.02% WASO: COMB 57.97%, CBT 10.29%, melatonin 42.46%, placebo 0.07% SE: COMB 20.00%, CBT 11.26%, melatonin 15.46%, placebo 1.12% 10.34% of the CBT only group attained SE of 85% or over and SOL of less than 30 min. None of the participants in the placebo group met either of these criteria	*r* = 0.67, *p* < 0.001 *r* = 0.61, *p* < 0.001 *r* = 0.53, *p* < 0.001 *r* = 0.62, *p* < 0.0001
Georén et al. (2022)	SE, SOL, TST, WASO and EMA	AIS, ISI and sleep diaries	Baseline, post‐intervention, 3 months, 6 months (AIS and ISI only for 3 and 6 months)	Improvements in all parameters at pre‐post intervention and FU, except EMA: Large reduction in mean scores on AIS and ISI from pre‐intervention to post‐intervention and small reduction from post‐intervention to 6‐month FU Large reduction in SOL from pre‐post intervention and large reduction in WASO from pre‐post; large increase in TST pre‐post intervention and large increase in SE pre‐post Moderate increase in EMA from pre‐post intervention Follow‐up or significance data for sleep diaries N/R	AIS: *d* = 1.71, ISI: *d* = 2.12 AIS: *d* = 0.20, ISI: *d* = 0.35 *d* = 0.82 *d* = 1.21 *d* = 1.76 *d* = 1.17 *d* = −0.53
Hiscock et al. (2019)	Sleep problem prevalence at 3 months	Parent‐report	Baseline, 3 months, 6 months	Intervention group reported less moderate/severe sleep problems than TAU group at 3 months (28.0% vs. 55.4%, *p* < 0.001) and 6 months (35.8% vs. 60.1%, *p* < 0.001)	Risk ratio (RR): 0.51, 95% CI 0.37, 0.70, *p* < 0.001 RR: 0.58, 95% CI 0.45, 0.76, *p* < 0.001
Ishii et al. (2021)	Insomnia severity	ISI and sleep diaries	Baseline, 5 weeks, 3 months	Large improvements in insomnia severity at post‐intervention Moderate improvement from pre‐intervention to 3‐month FU However, improvements post‐intervention to FU not sustained N/R for sleep diary data	*d* = 1.30, 95% CI 0.31, 2.28, *p* = 0.003 *d* = 0.41, 95% CI −0.48, 1.30, *p* = 0.035 *d* = −0.89, 95% CI −1.82, 0.04, *p* = 0.002
Jernelöv et al. (2019)	Insomnia severity	ISI	Baseline, 10 weeks, 3 months	Improvements in insomnia severity from pre‐post intervention and pre‐intervention to 3‐month FU; No significant results for post‐intervention to FU	*d* = 0.84, 95% CI 0.31, 1.37, *p* < 0.001 *d* = 1.52, 95% CI 0.87, 2.18, *p* < 0.001 *d* = 0.42, 95% CI −0.12, 0.95, *p* = 0.11
Lawson et al. (2023)	SE, SOL, TST, WASO and insomnia severity	Actigraphy, ISI and sleep diary	Questionnaires: baseline, 5 weeks, 2 months Actigraphy: 1 week prior to intervention and 1 week post‐intervention Sleep diary: baseline, 1 week post intervention and 1 week at follow‐up	Reduction in insomnia severity on ISI from pre‐ to post‐intervention, pre‐intervention to FU and post‐intervention to FU, however, the latter two results failed to achieve adjusted clinical significance Actigraphy data pre‐post intervention failed to reach statistical significance for all participants	*r* = −0.632, *p* = 0.011 *r* = −0.591, *p* = 0.027 *r* = −0.406, *p* = 0.129 SE: *r* = 0.03–*r* = 0.51, *p* > 0.001 SOL: *r* = −0.19–*r* = −0.41, *p* > 0.001 TST: *r* = 0.00–*r* = 0.49, *p* > 0.001 WASO: *r* = −0.03–*r* = 0.45, *p* > 0.001
McCrae et al. (2020)	SE, SOL, TST and TWT	Actigraphy and sleep diaries	Baseline, 8 weeks, 1 month follow‐up	Large objective improvements in SOL (−18 min), TWT (−34 min), and SE (+5%) data pre‐post intervention Large subjective improvements in SOL (−29 min), TWT (−45 min), TST (+63 min), and SE (+8%) pre‐post intervention However, slight decrease in improvements from post‐intervention to 1‐month follow‐up on actigraphy (e.g., SL: *M* post = 14.92 min, *M* FU = 15.73 min; TWT: *M* post = 72.20 min, *M* FU = 77.19 min; TST: *M* post = 545.72 min, *M* FU = 544.10 min; SE: *M* post = 85.81%, *M* FU = 84.78%) At post‐treatment, 92% of participants who completed treatment and assessment no longer met insomnia criteria. Proportion reduced to 85% at FU	SOL: *g*_av_ = 1.07, *p* = 0.01; TWT: *g*_av_ = 1.20, *p* = 0.00; TST: *g*_av_ = 0.87, *p* = 0.02 (not significant); SE: *g*_av_ = 1.22, *p* = 0.001 SOL: *g*_av_ = 1.41, *p* = 0.01; TWT: *g*_av_ = 1.33, *p* = 0.01; TST: *g*_av_ = 1.17, *p* = 0.001; SE: *g*_av_ = 2.15, *p* = 0.00 No ES provided for post‐intervention to FU
McCrae et al. (2021)	SE, SOL, TST and TWT	Actigraphy and sleep diaries	Baseline, 8 weeks, 1 month follow‐up	Large objective improvements in SOL (−17 min), TWT (−32 min), TST (+34 min), and SE (+5%) data pre‐to post‐intervention Large subjective improvements in SOL (−33 min), TWT (−46 min), TST (+52 min), and SE (+8%) pre‐ to post‐intervention Minor improvements in subjective TWT (*M* post = 23.19 min, *M* FU = 19.66 min), TST (*M* post = 575.95, *M* FU = 580.20), and SE (*M* post = 95.8%, *M* FU = 96.46%) from post‐intervention to FU, however, not confirmed by objective data apart from SE (*M* post = 84.56%, *M* FU = 85.16%) At post‐treatment, 92% of participants who completed treatment and assessment no longer met insomnia criteria. Proportion reduced to 83% at FU.	SOL: *g*_av_ = 1.10, *p* = 0.001; TWT: *g*_av_ = 0.97, *p* = 0.00; TST: *g*_av_ = 1.10, *p* = 0.00; SE: *g*_av_ = 1.08, *p* = 0.001 SOL: *g*_av_ = 1.45, *p* = 0.01; TWT: *g*_av_ = 1.36, *p* = 0.01; TST: *g*_av_ = 1.05, *p* = 0.001; SE: *g*_av_ = 1.97, *p* = 0.00 No ES provided for post‐intervention to FU

*Note*: Effect sizes: *g*_av_ = Hedges g, *d* = Cohen's *d*, *r* = Pearson correlation coefficient.

Abbreviations: AIS, Athens Insomnia Scale; EMA, early morning awakenings; N/R, not reported; SE, sleep efficiency; TST, total sleep time; TWT, total wake time.

### 3.8 Outcomes of CBT‐I Interventions on Mental Health, Sleep Habits and NDC Characteristics

A multitude of secondary outcomes were explored across studies, which mainly included mental health, sleep habits and severity of NDC characteristics. Given the positive impact of CBT‐I on other domains of well‐being in the typically developing population (e.g., Itani et al. 2017; Sadler et al. 2018; Scott et al. 2021), it is important to investigate whether the same applies for individuals with NDC.

Surprisingly few improvements were found in any of the secondary outcomes measured. Three studies assessed changes in quality of life, anxiety and depression (Hiscock et al. 2019; Ishii et al. 2021; Lawson et al. 2023), but only Lawson et al. (2023) observed improvements in anxiety (λ^2^ = 8.40, *p* = 0.015). However, those improvements were no longer statistically significant following Wilcoxon signed‐rank testing. Cortesi et al. (2012) reported minor improvements in sleep habits for the CBT‐I only group (0.6%). However, these improvements were larger for both the melatonin only group (17.83%) and the combined CBT‐I and melatonin group (27.83%). Hiscock et al. (2019) observed further improvements in sleep habits from baseline to follow‐up, but these were not sustained at 3‐ or 6‐month follow‐up.

Effects of CBT‐I interventions on NDC characteristics were the most promising. Four of five studies that assessed the severity of disorder‐specific characteristics reported improvements on at least one subscale (Ishii et al. 2021; Jernelöv et al. 2019; McCrae et al. 2020, 2021). Ishii et al. (2021) found that attention switching was the only ASD characteristic to improve from pre‐ to post‐intervention (*d* = 1.16, 95% CI 0.19, 2.13, *p* = 0.031). No other ASD or ADHD characteristic was significantly affected by time, and neither were total ASD and ADHD scores on the relevant scales. Jernelöv et al. (2019) reported a small reduction in total ADHD characteristics (*d* = 0.34, 95% CI 0.05, 0.62, *p* < 0.05) and improvements in the inattention subscale (*d* = 0.31, 95% CI 0.05, 0.57, *p* < 0.05) when comparing pre‐intervention scores with 3‐month follow‐up scores. McCrae et al. (2020) described reduced irritability (*g*_av_ = 0.83), lethargy (*g*_av_ = 0.56), stereotypy (*g*_av_ = 1.30) and hyperactivity (*g*_av_ = 0.42) in their sample of children with ASD following CBT‐I. All changes, apart from effects on hyperactivity, were maintained at 1‐month follow‐up. Similarly, in McCrae et al. (2021) hyperactivity did not improve at 1‐month follow‐up, but inappropriate speech (*g*_av_ = 0.80), irritability (*g*_av_ = 0.75), lethargy (*g*_av_ = 0.62) and stereotypy (*g*_av_ = 1.41) did.

Overall, based on the outcomes from studies included in this review, CBT‐I did not improve mental health or quality of life in people with NDC. Moreover, whilst positive changes in sleep habits were observed, Hiscock et al.'s (2019) results suggested no long‐term effectiveness. Effects of CBT‐I interventions on NDC characteristics were varied, with some describing no impact (Hiscock et al. 2019) and others reporting medium to large effects on ASD characteristics that appeared sustainable over time (McCrae et al. 2020, 2021) (see Table 4 for more details).

**TABLE 4:** Key secondary outcomes.

Author (year)	Secondary outcome measures	Method of assessment	Assessment points	Main outcomes	Effect sizes
Cortesi et al. (2012)	Bed resistance, SOD, sleep anxiety, night‐wakings, sleep duration, parasomnias, SBD, DS	Actigraphy, CSHQ	Baseline, 12 weeks	Large improvements of total sleep habit scores on CSHQ for melatonin + CBT (27.83%) and melatonin only (17.83%) groups from baseline to post‐intervention; Only minor improvements for CBT only (0.6%) and placebo groups (0.09%) pre‐ to post‐intervention	*r* = 0.78, *p* < 0.001
Hiscock et al. (2019)	Sleep difficulties, ADHD characteristics, behavioural difficulties, irritability, school attendance, quality of life, memory academic functioning, ASD, sleep hygiene	CSHQ, teacher daytime sleepiness questionnaire, ADHD rating scale IV, strengths and difficulties questionnaire, ARI, CHU‐9D, PedsQL, sleep suite app, working memory test battery for children	Baseline, 3 months, 6 months (sleep hygiene, PedsQL, CHU‐9D only at baseline and 6 months)	Improvements in sleep habits as measured by total CSHQ scores at 3‐month and 6‐month FU from baseline: pre‐intervention to 3 months: *M* = −4.20, *p* < 0.001, 95% CI 6.54, −1.86; pre‐intervention to 6 months: *M* = −3.70, *p* < 0.001, 95% CI 5.94, −1.46; no improvements from 3‐month FU to 6‐month FU No other significant results on secondary outcomes found, including ASD/ADHD characteristics, behavioural difficulties, quality of life, or academic functioning	No ES provided
Ishii et al. (2021)	ADHD, anxiety, depression	HADS, ASRS, AQ	Baseline, 5 weeks, 3 months	No significant results obtained for any secondary outcomes, except for a large effect on the Autism‐Spectrum Quotient attention switching subscale pre‐ to post‐intervention and small improvements pre‐intervention to FU; however, positive changes not sustained from post‐intervention to FU	*d* = 1.16, 95% CI 0.19, 2.13, *p* = 0.011 *d* = 0.33, 95% CI −0.56, 1. 22 *d* = − 0.70, 95% CI −1.61, 0.21
Jernelöv et al. (2019)	ADHD	ASRS	Baseline, 10 weeks, 3 months	Small effects on total ASRS scores assessing overall ADHD characteristic severity from baseline to 3‐month FU, but no significant effects on total scores at any other assessment point; small effects for pre‐intervention to FU on inattention subscale; small improvements on hyperactivity subscale pre‐ to post‐intervention; moderate improvements on hyperactivity subscale pre‐intervention to FU No significant results for any scales or subscales from post‐intervention to FU	*d* = 0.34, 95% CI 0.05, 0.62, *p* < 0.05 *d* = 0.31, 95% CI 0.05, 0.57, *p* < 0.05 *d* = 0.28, 95% CI 0.00, 0.55, *p* < 0.05 *d* = 0.44, 95% CI 0.20, 0.69, *p* < 0.001
Lawson et al. (2023)	Anxiety and depression	HADS PSQI CORE‐10 BEAQ SAAQ FFS	Baseline, 5 weeks, 2 months	Initial improvements in anxiety scores, however, following Wilcoxon signed‐rank test, results failed to meet statistical significance at pre‐ to post‐intervention, pre‐intervention to FU, and post‐intervention to FU No improvements in depression, fatigue, or overall distress	*λ* ^2^ = 8.40, *p* = 0.015 *r* = −0.553, *p* = 0.027 *r* = −0.54, *p* = 0.043 *r* = −0.257, *p* = 0.336
McCrae et al. (2020)	Challenging behaviours (irritability, lethargy, stereotypy, hyperactivity, inappropriate speech, bed/wake variabilit)y	Daytime functioning—aberrant behaviour checklist, actigraphy	Baseline, 8 weeks, 1 month follow‐up	Amelioration of a variety of challenging behaviours typical for ASD that were largely sustained at FU, except for hyperactivity: Moderate improvements pre‐ to post‐intervention for hyperactivity and lethargy; large improvements pre‐ to post‐intervention in irritability and stereotypy; no significant improvements for inappropriate speech at post‐treatment Improvements in hyperactivity not sustained from pre‐intervention to FU; large improvements from pre‐intervention to FU on inappropriate speech stereotypy, and irritability; moderate improvement pre‐intervention to FU on lethargy No effect sizes provided from post‐intervention to FU	*g*_av_ = 0.42, *p* = 0.001 *g*_av_ = 0.56, *p* = 0.00 *g*_av_ = 0.83, *p* = 0.00 *g*_av_ = 1.30, *p* = 0.00 *g*_av_ = 0.69, *p* = 0.01 *g*_av_ = 0.42, *p* = 0.01 *g*_av_ = 0.78, *p* = 0.00 *g*_av_ = 1.73, *p* = 0.00 *g*_av_ = 0.85, *p* = 0.00 *g*_av_ = 0.55, *p* = 0.00
McCrae et al. (2021)	Challenging behaviours (irritability, lethargy, stereotypy, hyperactivity, inappropriate speech, bed/wake variability)	Daytime functioning—aberrant behaviour checklist, actigraphy	Baseline, 8 weeks, 1 month follow‐up	Improvements in a multitude of challenging behaviours, similar to effects reported in McCrae et al. (2020): Large improvements from pre‐ to post‐intervention in irritability and stereotypy; medium improvements from pre‐ to post‐intervention in lethargy and hyperactivity; no significant improvements for inappropriate speech at post‐treatment Improvements in hyperactivity not sustained from pre‐intervention to FU; large improvements sustained for stereotypy at pre‐intervention to FU and large decrease in inappropriate speech from baseline to FU; moderate to large positive changes in lethargy and irritability No effect sizes provided from post‐intervention to FU	*g*_av_ = 0.86, *p* = 0.00 *g*_av_ = 1.16, *p* = 0.00 *g*_av_ = 0.64, *p* = 0.00 *g*_av_ = 0.58, *p* = 0.001 *g*_av_ = 0.72, *p* = 0.01 *g*_av_ = 0.40, *p* = 0.01 *g*_av_ = 1.14, *p* = 0.00 *g*_av_ = 0.80, *p* = 0.00 *g*_av_ = 0.62, *p* = 0.00 *g*_av_ = 0.75, *p* = 0.00

Abbreviations: AQ, Autism‐Spectrum Quotient; ARI, Affective Reactivity Index; ASRS, Adult ADHD Self‐Report; BEAQ, Brief Experiential Avoidance Questionnaire; CHU‐9D, Child Health Utility‐9D; CORE‐10, Clinical Outcomes in Routine Evaluation; CSHQ, Children's Sleep Habits Questionnaire; DS, Datetime Sleepiness; FFS, Flinders Fatigue Scale; HADS, Hospital Anxiety and Depression Scale; PedsQL, Paediatric Quality of life Inventory; PSQI, Pittsburgh Sleep Quality Index; SAAQ, Sleep Anticipatory Anxiety; SDB, Sleep Disordered Breathing; SOD, Sleep Onset Delay.

### 3.9 Quality Assessment and Risk of Bias

#### 3.9.1 Pre‐Post Studies

Most studies received a ‘fair’ rating (Georén et al. 2022; Ishii et al. 2021; Lawson et al. 2023; McCrae et al. 2020, 2021), whilst Jernelöv et al. (2019) achieved a rating of ‘good’. A major concern with all studies lies in their non‐randomised nature, which severely restricts the ability to decipher any causal effects of the intervention from confounding variables. Other main concerns relate to inconsistencies in enrolment, blinding and drop‐out rates. Lack of blinding across interventions may significantly increase detection bias and skew findings. Additionally, none of the studies adopted an interrupted time‐series design, thus increasing the chance of outcomes being less robust due to random fluctuations (Ewusie et al. 2020) (see Table 5 for more details).

**TABLE 5:** Quality assessment of pre–post studies using NHLBI (2021) tool.

Assessment questions	Study reference					
	Georén et al. (2022)	Ishii et al. (2021)	Jernelöv et al. (2019)	Lawson et al. (2023)	McCrae et al. (2020)	McCrae et al. (2021)
Q1. Was the study question or objective clearly stated?	Y	Y	Y	Y	Y	Y
Q2. Were eligibility/selection criteria for the study population prespecified and clearly described?	Y	Y	Y	N	Y	Y
Q3. Were the participants in the study representative of those who would be eligible for the test/service/intervention in the general or clinical population of interest?	Y	Y	Y	Y	Y	Y
Q4. Were all eligible participants that met the prespecified entry criteria enrolled?	N	N	Y	N	N	N
Q5. Was the sample size sufficiently large to provide confidence in the findings?	N	N	Y	N	Y	Y
Q6. Was the test/service/intervention clearly described and delivered consistently across the study population?	Y	N	N	Y	Y	Y
Q7. Were the outcome measures prespecified, clearly defined, valid, reliable and assessed consistently across all study participants?	Y	Y	Y	Y	N	N
Q8. Were the people assessing the outcomes blinded to the participants' exposures/interventions?	N	N	N	N	Y	Y
Q9. Was the loss to follow‐up after baseline 20% or less? Were those lost to follow‐up accounted for in the analysis?	Y	Y	Y	N	N	N
Q10. Did the statistical methods examine changes in outcome measures from before to after the intervention? Were statistical tests done that provided *p* values for the pre‐to‐post changes?	N	Y	Y	Y	Y	Y
Q11. Were outcome measures of interest taken multiple times before the intervention and multiple times after the intervention (i.e., did they use an interrupted time‐series design)?	N	N	N	N	N	N
Q12. If the intervention was conducted at a group level (e.g., a whole hospital, a community, etc.) did the statistical analysis take into account the use of individual‐level data to determine effects at the group level?	N/A	N/A	N/A	N/A	N/A	N/A

*Note*: Y = yes, *N* = no.

Abbreviation: N/A, not applicable.

#### 3.9.2 Randomised Controlled Trials

**FIGURE 2:**
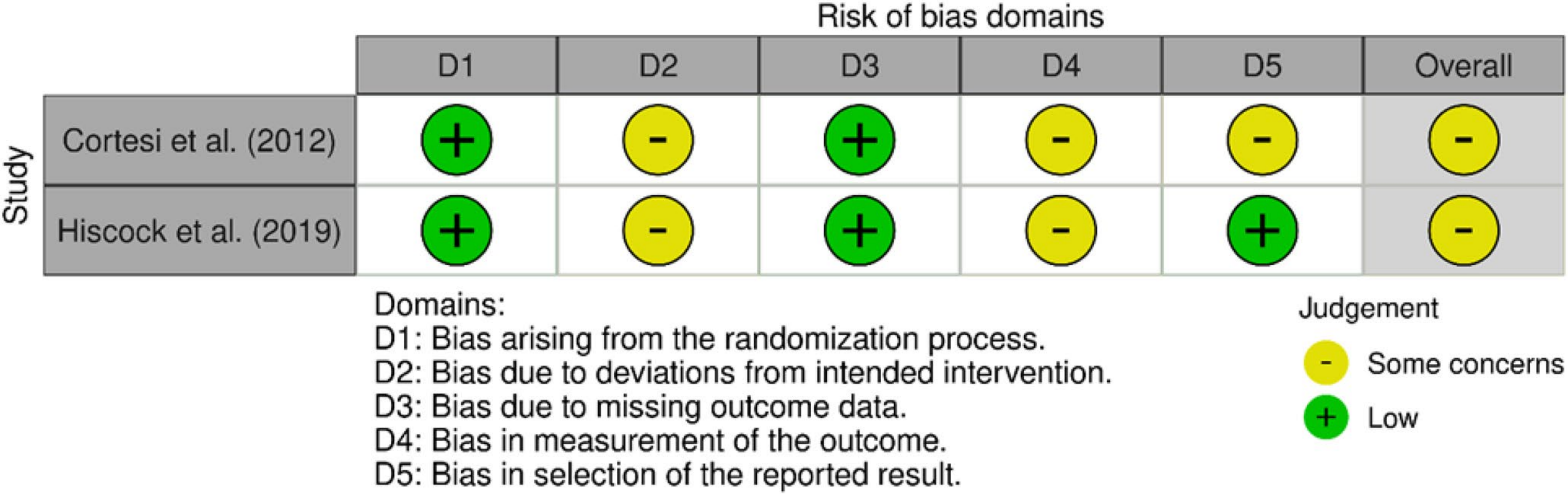
Visualisation of quality assessment of randomised controlled trials using RoB 2 (2019) tool. Web app ‘robvis’ (McGuiness and Higgins 2020) was used to create traffic light plot displaying risk of bias across five individual domains and overall rating.

Both RCTs received a rating between ‘low’ and ‘high’ risk of bias as there were ‘some concerns’, particularly regarding bias in outcome measurement and adherence to the intended intervention. No protocol could be found for the Cortesi et al. (2012) study, hindering a fair assessment of whether the trial was delivered, analysed and reported as intended and therefore increasing the risk of reporting bias. Additionally, a lack of follow‐up data means that long‐term effectiveness of the intervention is unknown. In Hiscock et al. (2019), the prevalence of sleep problems was chosen as the primary outcome of interest, and reporting was divided into dichotomous categories of none/mild and moderate/severe. This reporting fails to capture nuance in the data that is necessary for a comprehensive understanding of treatment effectiveness and less bias in outcome measurement. Additionally, not all individuals received their assigned treatment, with some being removed after randomization. This leads to concerns about whether the treatment was delivered as intended to all participants. Further, it was unclear whether individuals with missing data were excluded from the analysis (see Figure 2 for more details).

## 4 Discussion

### 4.1 Summary of Findings

This systematic review evaluated the effectiveness of CBT‐I for ID in individuals with NDC. Given the vast evidence based on the usefulness of CBT‐I in neurotypical populations (Trauer et al. 2015), including those with psychiatric and medical co‐morbidities (Hertenstein et al. 2022; Scott et al. 2021; Wu et al. 2015; Zhou et al. 2020), large and consistent findings were expected. However, the literature search provided few results and only two studies were RCTs. A lack of high‐quality CBT‐I based intervention research in those with NDC is apparent and has previously been reported (Esbensen and Schwichtenberg 2016; Pattison et al. 2020; Rigney et al. 2018).

Of the studies included in this review, most found modest improvements in insomnia severity, which were not maintained at follow‐up. Interestingly, one large RCT (Cortesi et al. 2012) found that a combination treatment of melatonin and CBT‐I, and treatment with melatonin only, yielded larger improvements than a CBT‐I intervention alone in their sample of children with ASD. This observation is supported by findings of other reviews (Cortese et al. 2020; Williams Buckley et al. 2020). Within the NDC population, the mechanisms underlying melatonin secretion and their impact on sleep and circadian rhythms are complex and varied (Woodford et al. 2021). Lower levels of melatonin, anomalous melatonin release throughout the day and night and associated severity of NDC characteristics have previously been reported in individuals with ASD (Benabou et al. 2017; Feybesse et al. 2023; Yenen and Çak 2020). Nonetheless, these processes are not yet fully understood, and more research is required to make definite statements and inform prospective sleep interventions (Woodford et al. 2021; Yenen and Çak 2020). Additionally, effects of CBT‐I on secondary outcomes (e.g., mental well‐being and severity of NDC characteristics) in the NDC population were unconvincing, despite previous research demonstrating the positive effects of CBT‐I on general well‐being (Alimoradi et al. 2022; Espie et al. 2019). However, occurrences of mental health problems were low at baseline, thus partly explaining an absence of large improvements in this domain.

In sum, interventions largely failed to mirror the success of standardised CBT‐I interventions in the neurotypical population. These findings may be due to the concerns raised in our quality assessment, such as very small sample sizes and moderate risk of bias. Moreover, the vast majority of CBT‐I research to date has been conducted with neurotypical adults. Within our review, most studies (*n* = 5) comprised child and adolescent samples, thus complicating direct comparisons of results. Further, a near absence of stakeholder and expert guidance on delivering CBT‐I to people with NDC led to a lack of consistency across trials. Nonetheless, it appears that the interventions that incorporated the most CBT‐I components also achieved some of the most positive outcomes (Georén et al. 2022; McCrae et al. 2020, 2021). These studies were also the ones that delivered the most CBT‐I sessions. Indeed, some authors (Ishii et al. 2021) noted that their intervention may have been too short to elicit the desired effects. Whilst four CBT‐I sessions have previously been recommended as most effective in treating ID using CBT‐I (Edinger et al. 2007), adaptations to this might be required for individuals with NDC. Such adaptations may include providing participants (and parents) with regular booster sessions to maintain acquired sleep habits and strategies, as this can be a particular challenge for people with NDC (Stores 2016). Booster sessions have previously been described as a likely requirement to increase long‐term effectiveness of CBT‐I (e.g., Sciberras et al. 2019). Mode of treatment delivery, whether self‐directed or delivered synchronously by professionals, does not seem to impact study outcomes (Georén et al. 2022; McCrae et al. 2021). Moreover, there were no significant differences between individual and group interventions. Previous comparisons, however, favour individual CBT‐I over group CBT‐I in typically developing adult populations (Yamadera et al. 2016).

A lack of clearly identifiable trends of differing effectiveness across treatments may have been influenced by the limited number of studies meeting inclusion criteria. Only three studies included adult samples (Ishii et al. 2021; Jernelöv et al. 2019; Lawson et al. 2023), emphasising that a large proportion of the research in this field is focussed on children (e.g., Cortese et al. 2020; Rigney et al. 2018). No observations could be made as to whether children or adult interventions were more effective. Similarly, gender differences in insomnia symptoms and treatment response were not discernible from the data available. However, it is noteworthy that 75% of all participants were male. Whilst this proportion aligns with diagnostic rates and previous reviews (e.g., Phillips et al. 2020), it highlights the need for increased efforts to accurately diagnose girls and women with NDC and to include female participants in the NDC and ID research niche to meet the needs of this population and make informed treatment decisions. Additionally, we would like to draw attention to the exclusion of participants with intellectual disability from all studies included in this review. Intellectual disability frequently co‐occurs with other NDC such as ASD (Matson and Shoemaker 2009), so to increase confidence in the clinical significance of study findings, future trials should include people with co‐occurring intellectual impairments, adapting CBT‐I protocols accordingly.

### 4.2 Strengths and Limitations

To our knowledge, this is the first review of its kind and thus offers the strength of systematically addressing a critical gap in the insomnia research literature. Efforts were made to be as comprehensive and inclusive as possible, by including a diverse sample of participants and a broad range of CBT‐I outcomes. Our inclusion criteria allowed for a variety of intervention characteristics to be considered. However, it is unclear which individual intervention components, or adaptations made for the NDC population, proved most effective. This would have been helpful information, but it was inaccessible from the published material reviewed. Similarly, although our eligibility criteria were inclusive of all NDC to maximise the usefulness of this review, included studies consisted exclusively of individuals with ASD and/or ADHD, thus providing a narrower focus of the review than desired. Additionally, the limited number of eligible studies (only two RCTs and six before‐after studies), most with small sample sizes and at least some risk of bias concerns, naturally restrict the conclusions that can be drawn. Lastly, although conducting a meta‐analysis was not possible here, future reviews would benefit from this approach to quantify heterogeneity and provide unitary effect sizes.

### 4.3 Conclusions

Given the limited number of available research studies, and the considerable inconsistencies and risk of bias reported here, the effectiveness of CBT‐I for people with NDC remains unclear. Nonetheless, study findings do give reason to be hopeful that CBT‐I may offer some promise for the severity of ID and NDC characteristics, particularly in the short term. Given the high prevalence of ID in the NDC population, we have identified the need for rigorous research to develop a CBT‐I protocol for people with NDC. We strongly suggest that this research includes a range of NDC, including intellectual disability, and considers the unique attributes and needs of this population. This may best be achieved by directly involving people with NDC in the design of future interventions. Moreover, it is suggested that booster sessions are offered to increase the long‐term effectiveness of treatment and provide sustainable insomnia relief.

## Author Contributions

**Maja Cullen:** conceptualization, writing – original draft, methodology, visualization, writing – review and editing, formal analysis, data curation. **Stephanie McCrory:** conceptualization, writing – review and editing, supervision. **Gemma Hooman:** data curation, methodology, conceptualization. **Megan Coyle:** methodology, data curation, conceptualization. **Leanne Fleming:** supervision, writing – review and editing, conceptualization.

## Conflicts of Interest

The authors declare no conflicts of interest.

## Data Availability

Data sharing is not applicable to this article as no new data were created or analyzed in this study.

## Appendix A

### Full Search Strategy

#### Search Strategy Used in PsycInfo, Scopus, Medline and Google Scholar

1. insomnia* OR ‘sleep disturbance*’ OR ‘sleep disorder*’ OR sleeplessness OR ‘sleep problem*’ OR ‘behavio* sleep disorder*’ OR ‘DIMS, Disorders of Initiating and Maintaining Sleep’ OR ‘Disorders of Initiating and Maintaining Sleep’ OR ‘Early Awakening’ OR ‘Insomnia Disorder*’, OR ‘sleep fragmentation’ OR ‘frequent awakening*’ OR ‘sleep onset’ OR ‘late insomnia’ OR ‘sleep maintenance’ OR ‘primary insomnia’
2. neurodiver* OR ‘neurodevelopmental disorder*’ OR ‘neuropsychological disorder*’ OR ‘neuropsychology disabilit*’ OR ‘neurodisability’ OR ‘neurdevelopmental condition*’ OR ‘ASD’ OR Autis* OR ‘autism spectrum disorder*’ OR ASC OR ‘autism spectrum condition*’ OR ‘attention deficit hyperactivity disorder*’ OR ADHD OR ‘attention deficit disorder*’ OR ‘attention deficit*’ OR asperger* OR ‘intellectual disability*’ OR ‘learning difficult*’
3. Intervention* OR CBT OR ‘cognitive behavio* therap*’ OR ‘cognitve therap*’ OR ‘behavio* intervention*’ OR ‘sleep restriction’ OR ‘bed restriction’ or ‘sleep hygiene’ OR ‘relaxation’ OR ‘sleep education’ OR ‘stimulus control’ OR ‘cognitive intervention*’ OR ‘psychoeducation’

#### Search Strategy Used in Cochrane Library

1. insomnia* OR (sleep NEXT disturbance*) OR (sleep NEXT disorder*) OR sleeplessness OR (sleep NEXT problem*) OR (behavio* NEXT sleep NEXT disorder*) OR ‘DIMS, Disorders of Initiating and Maintaining Sleep’ OR ‘Disorders of Initiating and Maintaining Sleep’ OR ‘Early Awakening’ OR (Insomnia NEXT Disorder*), OR ‘sleep fragmentation’ OR (frequent NEXT awakening*) OR ‘sleep onset’ OR ‘late insomnia’ OR ‘sleep maintenance’ OR ‘primary insomnia’.
2. neurodiver* OR (neurodevelopmental NEXT disorder*) OR (neuropsychological NEXT disorder*) OR (neuropsychology NEXT disabilit*) OR ‘neurodisability’ OR (neurdevelopmental NEXT condition*) OR ‘ASD’ OR Autis* OR (autism NEXT spectrum NEXT disorder*) OR ASC OR (autism NEXT spectrum NEXT condition*) OR (attention NEXT deficit NEXT hyperactivity NEXT disorder*) OR ADHD OR (attention NEXT deficit NEXT disorder*) OR (attention NEXT deficit*) OR asperger* OR (intellectual NEXT disability*) OR (learning NEXT difficult*)
3. Intervention* OR CBT OR (cognitive NEXT behavio* NEXT therap*) OR (cognitve NEXT therap*) OR (behavio* NEXT intervention*) OR ‘sleep restriction’ OR ‘bed restriction’ or ‘sleep hygiene’ OR ‘relaxation’ OR ‘sleep education’ OR ‘stimulus control’ OR (cognitive NEXT intervention*) OR ‘psychoeducation’
